# Supplementary figures and images for: Neuroprotective Activity of Mentha Species on Hydrogen Peroxide-Induced Apoptosis in SH-SY5Y Cells
Source: Nutrients. 2020 May 10;12(5):1366. doi: 10.3390/nu12051366 (PMC7285141; doi:10.3390/nu12051366)

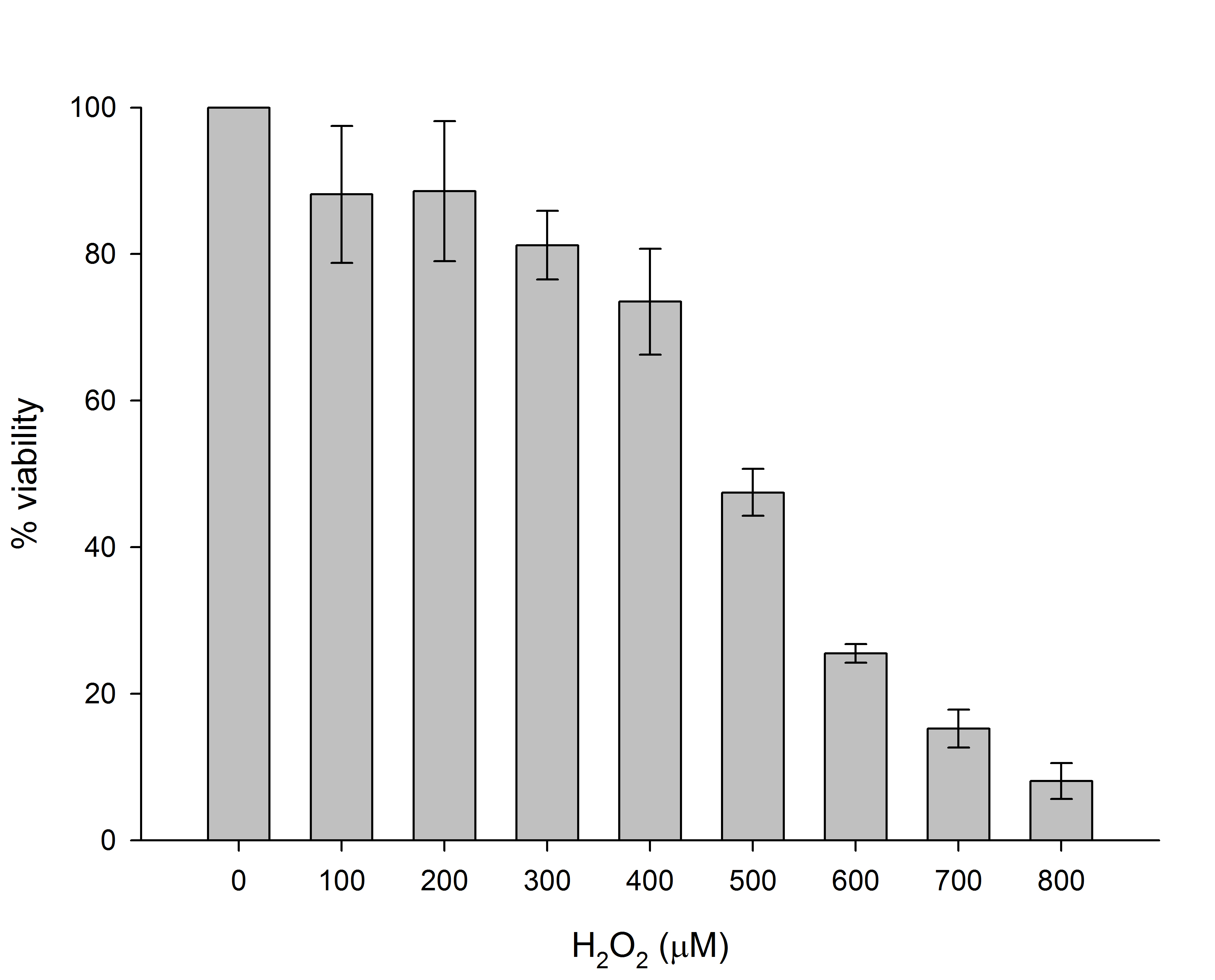

Supplement: Supplementary file 1 [file nutrients-12-01366-s001.zip › Figure S1.tif]

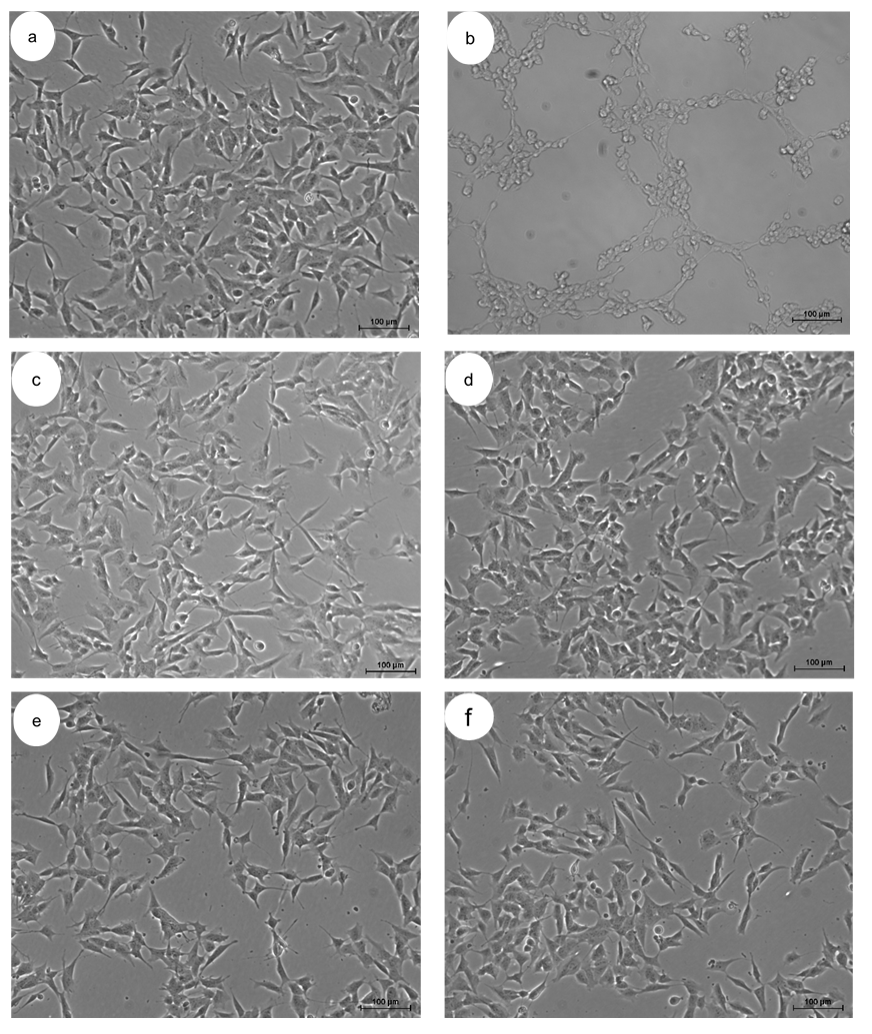

Supplement: Supplementary file 1 [file nutrients-12-01366-s001.zip › Figure S2_Nutrients.tif]

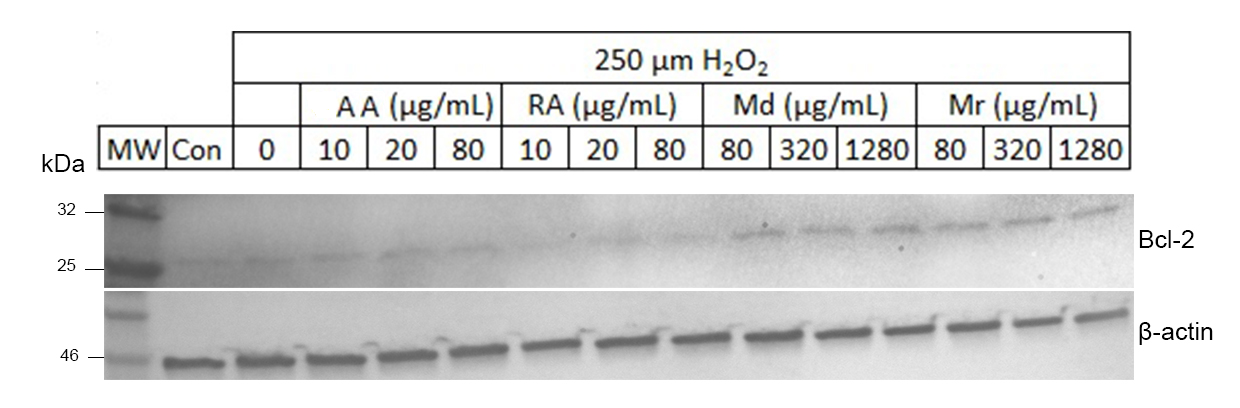

Supplement: Supplementary file 1 [file nutrients-12-01366-s001.zip › Figure S4.TIF]

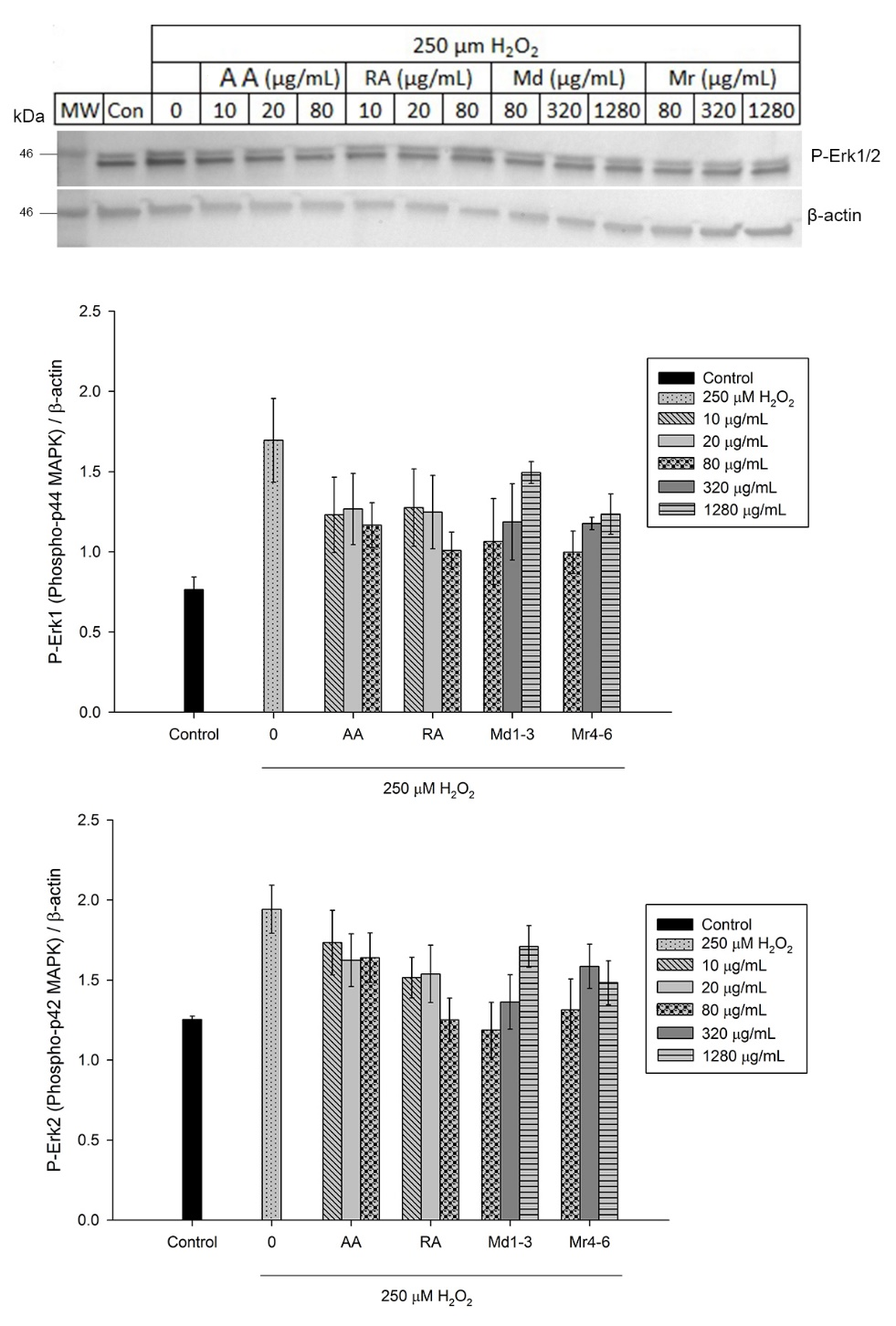

Supplement: Supplementary file 1 [file nutrients-12-01366-s001.zip › Figure S5.TIF]
